# Stabilization of benzene radical anion in ammonia clusters†

**DOI:** 10.1039/d2cp02979k

**Published:** 2022-11-02

**Authors:** Andriy Pysanenko, Stefan Bergmeister, Paul Scheier, Michal Fárník

**Affiliations:** J. Heyrovský Institute of Physical Chemistry, v.v.i., The Czech Academy of Sciences, Dolejškova 2155/3, 182 23 Prague Czech Republic michal.farnik@jh-inst.cas.cz +420 2 6605 3910 +420 2 6605 3206; Institut für Ionenphysik und Angewandte Physik, Universität Innsbruck, Technikerstr. 25 A-6020 Innsbruck Austria

## Abstract

We investigate electron attachment to large ammonia clusters doped with a single benzene (Bz) molecule (NH_3_)_*N*_·Bz, *N̄* ≈ 320. Negatively charged clusters are probed by mass spectrometry, and the energy-dependent ion yields are derived from mass spectra measured at different electron energies. The ion efficiency curves of pure ammonia clusters exhibit two maxima. At around 6 eV, (NH_3_)_*n*−1_NH_2_^−^ ions are produced *via* dissociative electron attachment (DEA) to NH_3_ molecules. (NH_3_)_*n*_^−^ ions produced at this energy are formed by DEA followed by fragment caging. At low energies around 1.3 eV, only (NH_3_)_*n*_^−^ ions are formed for cluster sizes *n* ≥ 35 that correspond to solvated electrons in ammonia clusters. The doped (NH_3_)_*n*_·Bz^−^ cluster ions exhibit essentially the same energy dependence. The (NH_3_)_*n*_·Bz^−^ ions are metastable and evaporate NH_3_ molecule(s), while pure (NH_3_)_*n*_^−^ ions are stable. The lifetime for NH_3_ molecule evaporation from the Bz-doped clusters was estimated as *τ* ≈ 18 μs. We interpret the metastability of the doped clusters by the charge localization on a Bz^−^ ion solvated in the ammonia, which is accompanied by an energy release leading to the evaporation of NH_3_ molecule(s).

## Abstract 2

Ammonia clusters are doped with benzene molecules in a pickup experiment, and slow electrons are attached to the pure and doped clusters. Generation of solvated electron and stabilization of a benzene radical anion is demonstrated.

## Graphical abstract

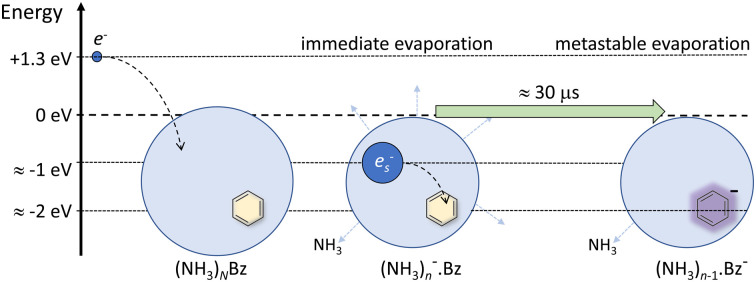

## 1 Introduction

The Birch reduction process^1^ is used in organic chemistry for reduction of aromatic compounds with a high level of control, *e.g.*, converting benzene to 1,4-cyclohexadiene. Benzene (Bz), ammonia (NH_3_) and solvated electrons (e_aq_^−^) are the essential ingredients in this process. Experiments with negatively charged clusters of ammonia doped with benzene molecules can provide a molecular-level insight into the stability, dynamics and chemistry of the excess electron (solvated electron) in such a system.

Negatively charged ammonia clusters (NH_3_)_*n*_^−^ were investigated experimentally since the early cluster beam experiments in Haberland's group.^2,3^ (NH_3_)_*n*_^−^ clusters were produced by injecting low energy electrons into supersonic expansions of NH_3_ gas. A minimum cluster size of *n* = 35 was observed independent of the expansion conditions. Later studies linked these clusters to solvated (ammoniated) electrons.^4,5^ Theory confirmed a sharp transition from unstable to stable (NH_3_)_*n*_^−^ clusters at around *n* ≈ 30 and predicted that smaller (NH_3_)_*n*_^−^ clusters with *n* ≈ 14–34 should exist, however, a large rearrangement was required to accept the excess electron.^6^ Further theoretical investigations studied weakly bound (NH_3_)_*n*_^−^ clusters and the dynamics of electron solvation.^7,8^

Small ammonia cluster ions (NH_3_)_*n*_H^−^ and (NH_3_)_*n*_NH_2_^−^ with *n* = 1 and 2 were investigated by photoelectron spectroscopy.^9,10^ Isolated ammonia molecules were also investigated with respect to the attachment of an excess electron. The dissociative electron attachment (DEA) of slow electrons to NH_3_ molecules can yield two products: H^−^ and NH_2_^−^. Both fragment ions are generated *via* two resonances at electron energies around 6 eV and at 10.5 eV.^11–14^

The intermediate species generated in ammonia solvent in the Birch reduction process is the benzene radical anion C_6_H_6_^−^ (Bz^−^). From the point of view of quantum chemistry it exhibits some intriguing properties; it is a stable anion in solutions,^15^ but it has the character of a metastable shape resonance in the gas phase.^16^ Benzene molecules have a negative electron affinity of EA = −1.15 eV. Nevertheless, large negatively charged benzene clusters (Bz)_*n*_^−^ were generated by slow electron attachment at sizes *n* ≥ 53.^17^ In the direct relevance to the Birch reduction, Kostal *et al.*^18^ have recently investigated the stabilization of the benzene radical anion in ammonia clusters theoretically. They observed a transition from an unbound electron on an isolated Bz molecule to a bound state of the benzene radical anion fully solvated in ammonia clusters (NH_3_)_*n*_Bz^−^. Here, we investigate the same species experimentally.

## 2 Experiment

The experiments were performed on our cluster beam apparatus (CLUB), which was described in reviews.^19,20^ In the present experiment the ammonia clusters are generated in a continuous supersonic expansion of pure ammonia gas through a conical nozzle (50 μm diameter, 30° full opening angle, and 2 mm long). The nozzle was kept at a constant temperature of *T*_0_ = 310 K and the stagnation pressure was *P*_0_ = 4 bar. These expansion conditions result in a mean (NH_3_)_*N*_ cluster size *N̄* ≈ 320 as determined from a semiempirical formula based on experimental measurements of Na-doped clusters.^21^ It is worth noting, that the original extensive measurements of Bobbert *et al.*^21^ were performed with our present cluster source and nozzle, thus we regard the neutral cluster size determination in our present experiment reliable.

The clusters pass through several differentially pumped vacuum chambers with background pressures below 10^−6^ mbar. The first chamber can be used to dope the clusters with benzene molecules. The pickup chamber is filled with benzene vapor at a given constant pressure *p*_Bz_ ≈ 2.5 × 10^−5^ mbar (the ionization gauge pressure value has been corrected by the gas correction factor of 5.90 for benzene). The cluster beam passes through the chamber on a path-length of *L* = 17 cm. Assuming a mean cluster size of *N̄* ≈ 320, and the ammonia molecules generating a sphere with a density corresponding to that of solid ammonia *ρ* = 817 kg *m*^−3^, we can derive a mean cluster radius *R̄* ≈ 1.4 nm. Consequently, a cluster approximated by a solid sphere with a radius *R̄* will collide with *k* = *σ*·*n*_Bz_·*L* molecules in the pickup cell, where *σ* = π(*R̄* + *r*_Bz_)^2^ is the cluster collision cross section with benzene molecules of radius *r*_Bz_ ≈ 0.28 nm, and *n*_Bz_ = *p*_Bz_/*k*_B_*T* is the benzene density in the pickup chamber. This simple estimate delivers *k* ≈ 0.9. Thus, assuming a sticking coefficient of one for Bz on (NH_3_)_*N*_, the ammonia clusters pick up about one benzene molecule on average. We perform our experiments under these weak doping conditions, since our aim is to observe the behavior of an excess electron in (NH_3_)_*n*_Bz^−^ clusters with a single Bz molecule. Both, the mass spectra of negatively and positively charged ions confirm that the present pickup generates the doped clusters predominantly just with one Bz molecule.

After passing through two more differentially pumped vacuum chambers, the clusters enter a chamber hosting a reflectron time-of-flight mass spectrometer (TOF). The TOF was first implemented and described elsewhere.^22,23^ It can detect either positive ions as described in the above cited publications, or it can work in the negative ion mode.^24,25^ The clusters are ionized by an electron beam pulsed at 10 kHz frequency. For positive ions, the clusters were ionized by 70 eV electrons. The ionization pulse width was 3 μs. After 0.1 μs delay, the ions were extracted by a 4 kV pulse and further accelerated to 8 keV into the time-of-flight region. In the negative ion mode, the electron pulse width and delay were 5 μs and 0.1 μs, respectively, and the extraction and acceleration voltages were 4 kV and 8 kV respectively. The electron energy was scanned between 0 and 14 eV in steps of 0.2 eV. From the mass spectra recorded at different electron energies, the energy dependent negative ion yield was evaluated. The electron energy scale was calibrated using the 4.4 eV and 8.2 eV resonances in dissociative electron attachment to CO_2_ molecules. It ought to be mentioned that our electron gun was originally designed for working with higher electron energies, and thus the energy dependence at electron energies below ≈1.5 eV is less reliable due to a low electron current caused by the electron beam defocusing. After the flight path of approximately 95 cm in the reflectron TOF the ions are detected with a microchannel plate detector and the mass spectra are recorded.

It should be noted that our mass scale has been calibrated to exact masses and not just to integer values; *i.e.*, the mass of an ammonia molecule is *m*(NH_3_) = 17.02655 u rather than 17 u. It has to be considered when calculating the corresponding cluster ion *m*/*z* in the mass spectrum especially for large clusters.

## 3 Results

### 3.1 Pure ammonia clusters

**Fig. 1:**
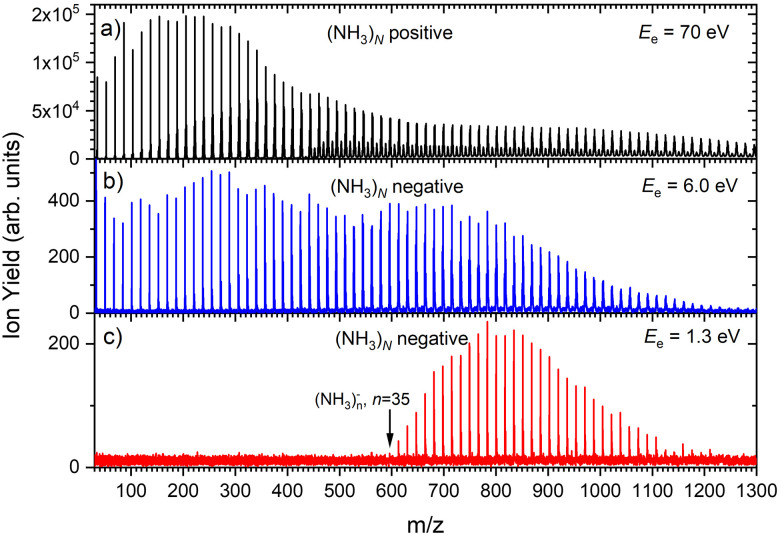
The mass spectra of pure ammonia clusters generated at *P*_0_ = 4 bar, *T*_0_ ≈ 310 K (*N̄* ≈ 320): (a) positive ions after 70 eV electron ionization; (b) negatively charged clusters after 6.0 eV electron attachment; (c) negatively charged clusters after 1.3 eV electron attachment.

Fig. 1 shows three mass spectra of pure (NH_3_)_*N*_ clusters with an average size of *N̄* ≈ 320. Detailed mass spectra with assignment of the main cluster ion series are given in ESI† (Fig. S1). The top panel (a) shows a positive ion spectrum after 70 eV electron ionization. It is dominated by protonated (NH_3_)_*n*_H^+^ cluster ions. From about *n* = 50 also doubly charged ions appear in the spectrum. Indeed, the mass spectrum does not correspond to the neutral cluster size distribution due to the cluster fragmentation after the ionization and also due to the mass discrimination caused by the perpendicular arrangement of our TOF mass spectrometer. These effects have been discussed in details in our previous publications.^26^Fig. 1(b) and (c) show the negatively charged cluster ion spectra upon attachment of electrons with a kinetic energy of 6.0 eV and 1.3 eV, respectively. The choice of these energies is clarified by the ion efficiency curve measurements below.

**Fig. 2:**
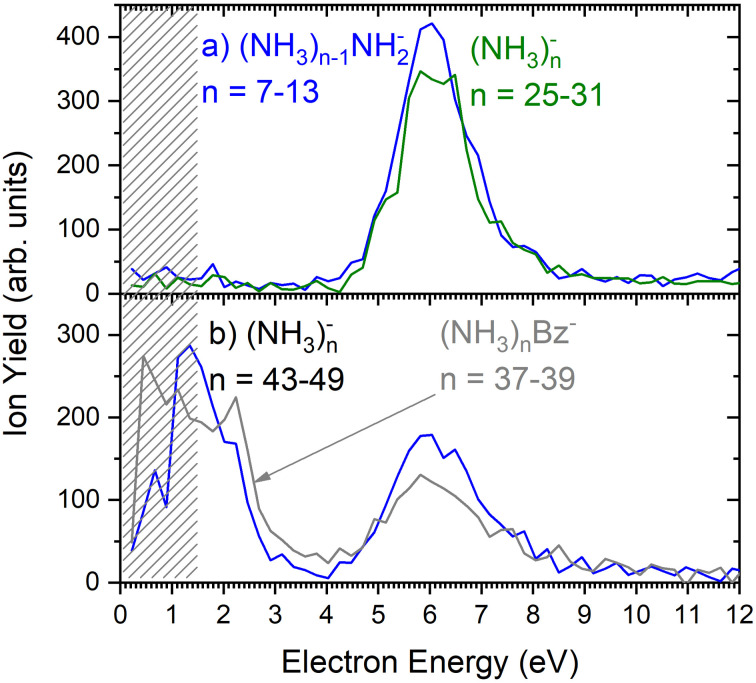
The negative ion yield dependence on the electron energy: (a) the integrated ion efficiency curves for (NH_3_)_*n*−1_NH_2_^−^ ions *n* = 7–13 (blue) and (NH_3_)_*n*_^−^, *n* = 25–31 (green); (b) the integrated ion efficiency curves for (NH_3_)_*n*_^−^ ions *n* = 43–49 (blue), and the doped (NH_3_)_*n*_Bz^−^, *n* = 37–39, clusters (grey). The shaded area below 1.5 eV indicates region, where the spectrum is less reliable as explained in the experimental section.

**Fig. 3:**
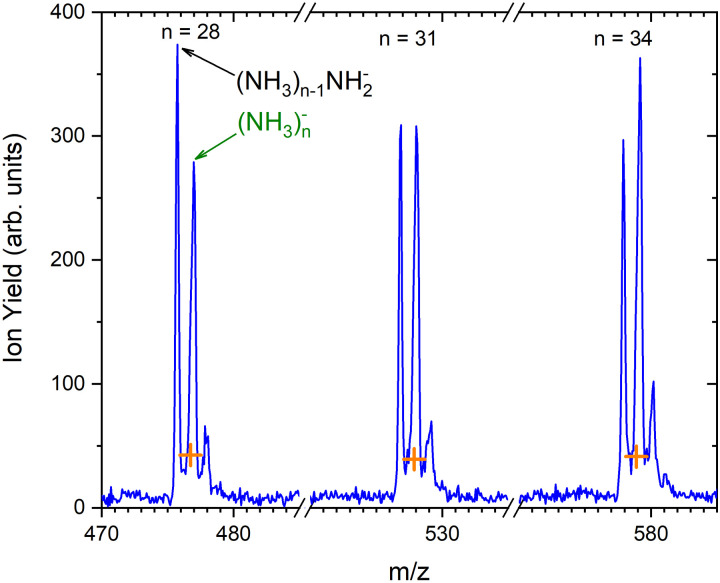
The detail of the mass spectrum showing the (NH_3_)_*n*−1_NH_2_^−^ and (NH_3_)_*n*_^−^ ion peaks for *n* = 28, 31 and 34. The orange crosses indicate the isotope contributions corresponding to the (NH_3_)_*n*−1_NH_2_^−^ isotope contribution.

As outlined in the Experimental section, the energy dependent ion yield is evaluated from the mass spectra measured at different electron energies for the negatively charged clusters. At low masses, the spectra are dominated by (NH_3_)_*n*−1_NH_2_^−^ ions. We integrate several mass peaks, for which the ion efficiency curves are essentially identical, to obtain a better signal-to-noise ratio. Fig. 2(a) shows the integrated ion yield of (NH_3_)_*n*−1_NH_2_^−^ ions between *n* = 7 and 13 (blue trace). At higher masses, (NH_3_)_*n*_^−^ ions appear. The abundance of (NH_3_)_*n*_^−^ ions integrated for sizes *n* = 25–31 is comparable with the (NH_3_)_*n*−1_NH_2_^−^, *n* = 7–13, ion yield (green line in Fig. 2a), and the (NH_3_)_*n*_^−^ ions dominate the spectrum with increasing *n*. The transition from the (NH_3_)_*n*−1_NH_2_^−^ to the (NH_3_)_*n*_^−^ ion dominated region of the mass spectra is shown in Fig. 3. The integrated ion yield of the (NH_3_)_*n*_^−^ ions from this region is also shown in Fig. 2(a) (green trace). It agrees well with the (NH_3_)_*n*−1_NH_2_^−^ ion yield, which suggests that the (NH_3_)_*n*_^−^ ions in this mass region are produced by the same mechanism as the (NH_3_)_*n*−1_NH_2_^−^ ones.

However, for the larger cluster ions where (NH_3_)_*n*_^−^ ions dominate the spectrum, the energy dependence exhibits a different character. This is demonstrated by the (NH_3_)_*n*_^−^ ion yield integrated for the peaks between *n* = 43 and 49 shown in Fig. 2(b). At low energies around 1.3 eV an additional maximum appears besides the one at 6 eV. It ought to be noted that 1.3 eV is already in the electron energy region, where the low electron current can cause an uncertainty in the maximum peak position. Thus, its exact position cannot be determined, nevertheless, the presence of a low-energy peak is unambiguous.

The 6 eV peak common to the (NH_3_)_*n*−1_NH_2_^−^ and (NH_3_)_*n*_^−^ ion efficiency curves is in good agreement with the dissociative electron attachment (DEA) cross section of gas phase ammonia molecules: at this energy, DEA to an NH_3_ molecule leads to H^−^ and NH_2_^−^ ion fragments.^13^ Thus, the (NH_3_)_*n*−1_NH_2_^−^ ions can be produced at around 6 eV by a DEA process in analogy to the DEA to isolated NH_3_ molecules. For sufficiently large *n* the (NH_3_)_*n*_^−^ ions can originate also from a DEA process, however, the departing H or H^−^ fragment is caged in large clusters. The stabilization of the transient negative ion (TNI) or a caging process was observed in quite a few cluster studies previously.^24,25,27,28^ The weaker maximum around 10.5 eV present in DEA to NH_3_ molecules,^13,29^ is not observed in our clusters. It is more than an order of magnitude weaker than the maximum at 6 eV for the NH_2_^−^ producing channel in the DEA to NH_3_ molecules.^13^ For the H^−^ producing channel, the maximum at 10.5 eV is about 4-times weaker than that at 6 eV, but it yields H^−^ fragments with high kinetic energies around 3 eV, which may leave the cluster.^29^

**Fig. 4:**
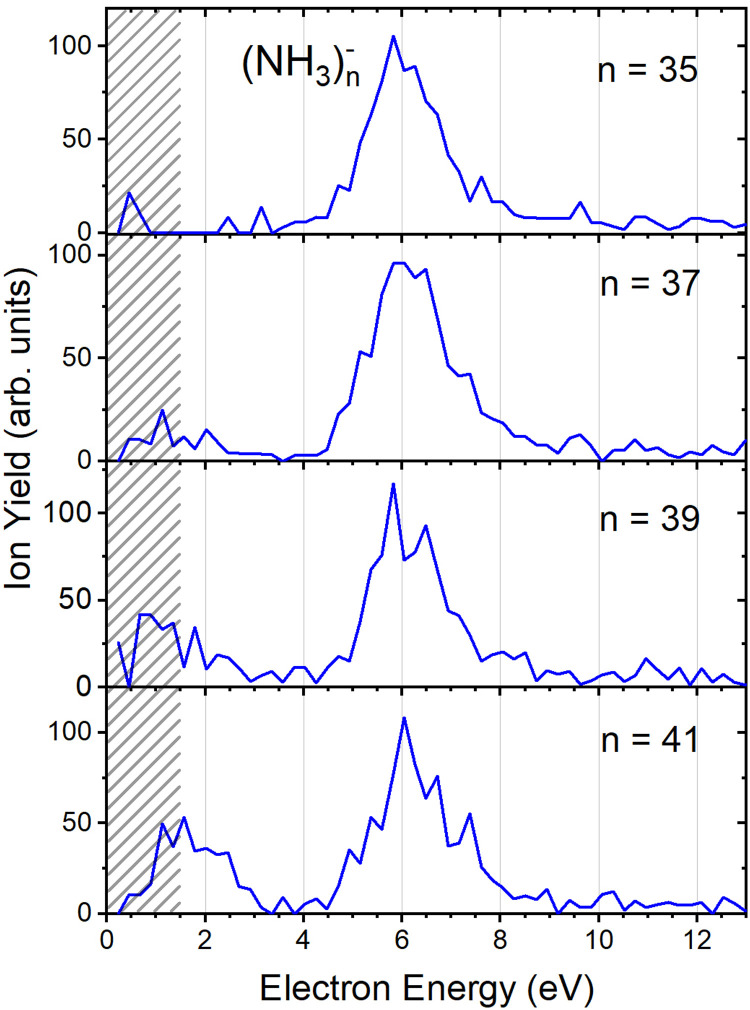
The negative ion yield dependence on electron energy for different (NH_3_)_*n*_^−^ ions between *n* = 35 and 41. The low-energy peak starts appearing for *n* = 37. The shaded area below 1.5 eV indicates a region of increased error bars as in Fig. 2.

Fig. 2(b) suggests that the larger (NH_3_)_*n*_^−^ cluster ions are produced by two different mechanisms. The electrons with energies around 6 eV can lead to DEA and subsequent caging as proposed above. Low energy electrons around 1.3 eV can lead to the formation of a solvated electron in ammonia clusters. The low energy peak starts appearing for (NH_3_)_*n*_^−^ ions with *n* ≥ 37 as shown in Fig. 4. This roughly corresponds to the threshold size for the low-energy electron attachment to ammonia clusters observed previously at *n* = 35,^2,3^ which was interpreted as being due to the formation of a solvated electron.^2–5^ Thus, the low-energy peak in the ion yield spectra corresponds to this process. It is also in agreement with our mass spectrum in Fig. 1(c) discussed below, where the threshold size *n* = 35 for the (NH_3_)_*n*_^−^ ions is observed as indicated. The mass spectrum in Fig. 1(c) corresponds to 10^8^ sweeps. On the other hand, the ion yield curves in Fig. 4 are derived from mass spectra corresponding to 10^6^ sweeps per one single energy. Therefore, the mass spectra in Fig. 1 exhibit a significantly better signal-to-noise ratio and the threshold size *n* = 35 for the (NH_3_)_*n*_^−^ ions is better determined. Nevertheless, the energy spectra support the assignment of the low energy peak to the formation of a solvated electron in ammonia clusters.

Based on the above negative ion yield energy dependencies, we have accumulated the mass spectra at two specific electron energies of 6 eV and 1.3 eV, Fig. 1(b) and (c), respectively. The 6 eV spectrum exhibits the above outlined trends: small ions are dominated by the fragmented species (NH_3_)_*n*−1_NH_2_^−^. From *n* = 6, these ions are accompanied by a mass peak displaced by Δ*m*/*z* = +1 corresponding to (NH_3_)_*n*_^−^ ions. At around *n* ≈ 30, the (NH_3_)_*n*_^−^ ions start to dominate the spectrum as shown in Fig. 3. There is also an isotope contribution of (NH_3_)_*n*−1_NH_2_^−^ ions to the Δ*m*/*z* = +1 peaks, however, it cannot explain the peaks as indicated in Fig. 3 by the orange crosses (see ESI† for calculation of the isotope contribution). In addition, there are mass peaks displaced by Δ*m*/*z* = +1 from the (NH_3_)_*n*_^−^ ions. They could correspond to (NH_3_)_*n*_H^−^ ions, since H^−^ is produced in the DEA of NH_3_^13^ and the (NH_3_)_*n*_H^−^, *n* = 1 and 2, ions were observed and investigated.^9^ However, there is also an isotope contribution of the (NH_3_)_*n*_^−^ cluster ions to the Δ*m*/*z* = +1 mass. A more detailed analysis is complicated by a metastable decay of the cluster ions in the TOF as will be discussed below. However, further analysis of the spectra in Fig. 1(c) presented below suggests that the contribution of (NH_3_)_*n*_H^−^ ions is negligible.

**Fig. 5:**
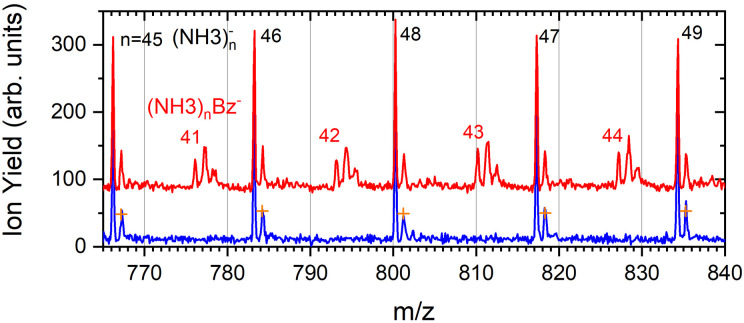
The detail of the negative mass spectra of ammonia clusters after pickup of benzene molecules measured at the electron energy of 1.3 eV (red). The spectrum of the pure ammonia clusters is shown for comparison (blue). The natural isotope contribution of (NH_3_)_*n*_^−^ ions is indicated by the orange crosses.

The mass spectrum recorded at 1.3 eV electron energy in Fig. 1(c) exhibits exclusively (NH_3_)_*n*_^−^ ions. There are small peaks displaced by Δ*m*/*z* = +1 from the (NH_3_)_*n*_^−^ ions, as shown by the bottom (blue) trace in Fig. 5. However, these are due to the isotope contribution as indicated by the orange crosses. Thus, no (NH_3_)_*n*_*H*^−^ ions are generated at 1.3 eV electron energy.

The above mentioned metastable decay of cluster ions in a TOF will be especially pronounced and important for benzene-doped clusters discussed in the next section. Thus, we provide a brief explanation here. A reflectron TOF can be used to investigate metastable decay reactions of the observed ions. For example, evaporation of individual molecules from positively charged ammonia and water clusters was studied previously with a reflectron TOF, and the method has been described in detail in ref. 30, 31 and references cited therein. Only a brief explanation is given here. If an (NH_3_)_*n*_NH_2_^−^ ion evaporates an NH_3_ molecule in the field-free region of our TOF before it enters the reflectron mirror, an (NH_3_)_*n*−1_NH_2_^−^ ion is reflected, and its signal does not appear in the mass spectrum at the mass corresponding to the (NH_3_)_*n*_NH_2_^−^ ion nor at the mass of (NH_3_)_*n*−1_NH_2_^−^. We can calculate the arrival times of stable and metastable ions in our TOF (see ESI,† for examples). At our present TOF settings, the (NH_3_)_*n*−1_NH_2_^−^ fragment originating from the metastable (NH_3_)_*n*_NH_2_^−^ ion decay appears very close to the position of the (NH_3_)_*n*_^−^ ion. Upon a close look, the peaks corresponding to the overlapping (NH_3_)_*n*_^−^ ions and the (NH_3_)_*n*−1_NH_2_^−^ fragments from the metastable decay are broader and their maximum is slightly shifted to higher values with respect to the exact (NH_3_)_*n*_^−^*m*/*z*. Thus, the peaks labeled as (NH_3_)_*n*_^−^ in Fig. 3 have three contributions: the stable (NH_3_)_*n*_^−^ ions, the fragments from (NH_3_)_*n*−1_NH_2_^−^ ← (NH_3_)_*n*_NH_2_^−^ metastable decay, and the isotope contribution of the stable (NH_3_)_*n*−1_NH_2_^−^ ions. Similarly, the next smaller peak following the (NH_3_)_*n*_^−^ ion contains the isotope contribution and also a contribution from the metastable decay of the (NH_3_)_*n*+1_NH_2_^−^ ion where two NH_3_ molecules are successively evaporated. A more detailed explanation and some examples for interested readers can be found in the ESI.†

### 3.2 Doped ammonia clusters

**Fig. 6:**
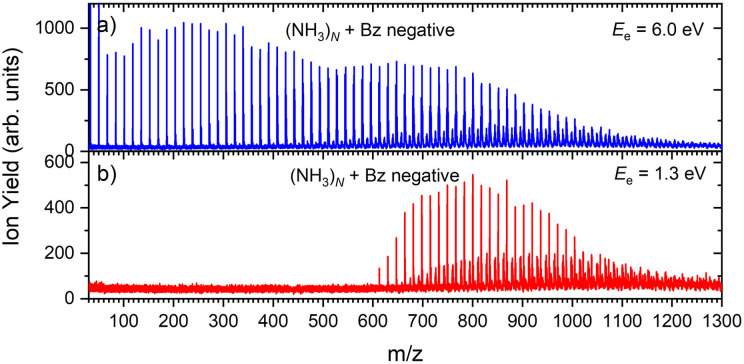
The negative mass spectra of ammonia clusters after pickup of benzene molecules measured at an electron energy of 6.0 eV (a) and 1.3 eV (b).

Fig. 6 shows two mass spectra of ammonia clusters after the pickup of benzene molecules as outlined in the Experimental section. Detailed spectra with assignment of the main cluster ion series are shown in ESI† (Fig. S2). The spectra were again measured at the electron energies of 6.0 eV (a) and 1.3 eV (b), and can be compared to the pure ammonia cluster spectra in Fig. 1(b) and (c), respectively. These energies are also confirmed by the ion efficiency curve for the benzene doped ion peaks (NH_3_)_*n*_Bz^−^ in Fig. 2(b) (grey line), which matches the shape of the pure clusters. In Fig. 6, there are new series arising with the pickup between the mass peaks corresponding to the pure ammonia clusters, which can be assigned to cluster ions containing a single benzene molecule. The clusters with just one benzene are confirmed also by a pressure dependence of positive cluster ion spectrum in ESI,† Fig. S3.

The series corresponding to the clusters with one Bz molecule is analogous to the series observed for pure ammonia clusters. In the top spectrum in Fig. 6(a) both (NH_3_)_*n*−1_NH_2_Bz^−^ and (NH_3_)_*n*_Bz^−^ are observed. In the bottom spectrum, Fig. 6(b), only the latter series is populated at 1.3 eV. However, multiple peaks are observed due to the metastable decay of the negatively charged clusters with benzene. A detailed section of the 1.3 eV spectrum is shown in Fig. 5 (red trace) together with the spectrum of the pure ammonia clusters for comparison (blue). After the pickup a group of three new peaks appears between the peaks corresponding to the (NH_3_)_*n*_^−^ ions.

**Fig. 7:**
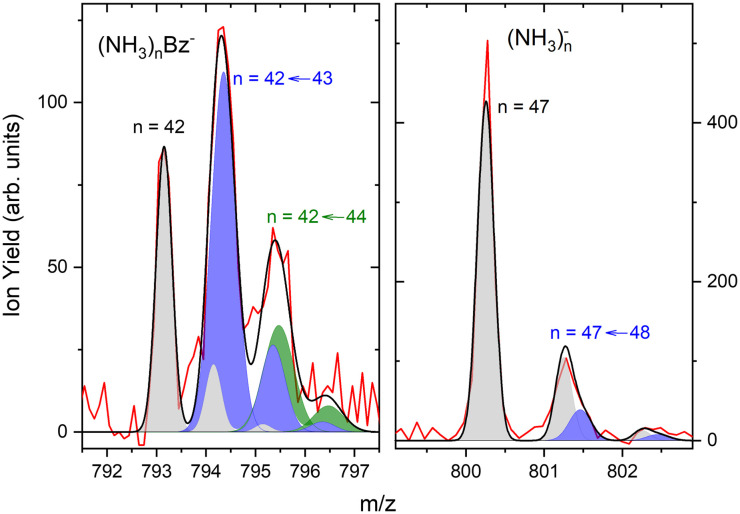
The detailed analysis of the mass spectrum peaks: (a) (NH_3_)_*n*_Bz^−^, *n* = 42 ion and its isotope contribution (grey), the peak from metastable decay *n* = 42 ← 43 with the corresponding isotope contribution (blue) and the corresponding peak from *n* = 42 ← 44 decay (green), sum of all peaks (black line) fitted to the experimental data (red); (b) (NH_3_)_*n*_^−^*n* = 47 peak (grey) with the metastable contribution (blue) within the noise limit.

The first sharp peak in the “triplet” always corresponds to the (NH_3_)_*n*_Bz^−^ ion. However, the accompanying peaks are broader and their positions do not quite correspond to any ion mass exactly, although their displacements from the first peak are close to Δ*m*/*z* = +1 and +2. These peaks are composed of several contributions as detailed in Fig. 7. In Fig. 7(a) the peak of (NH_3_)_*n*_Bz^−^, *n* = 42 is analyzed. This ion has an isotope contribution to the following masses displaced by Δ*m*/*z* = +1 and +2 of 23.8% and 2.7%, respectively. These contributions are indicated by the gray areas based on a Gaussian fit of the first (NH_3_)_42_Bz^−^ peak. Clearly, these peaks cannot account for the observed intensities. Therefore, a metastable decay of larger (NH_3_)_*n*_Bz^−^ ions is invoked as mentioned above. Namely, the evaporation of one NH_3_ molecule from *n* = 43 ion, and evaporation of two NH_3_ molecules from *n* = 44 yield the blue and green peaks, respectively, as indicated. If the evaporation happens in the first field-free TOF region the calculated positions correspond to the observed peaks. Thus, we fix the isotope contribution (grey) and fit the additional Gaussian peaks corresponding to the metastable cluster decays to the experimental data. Actually, four peaks are fitted, since each metastable peak has an accompanying isotope contribution. The intensity and position of the corresponding isotope peaks are tied to the metastable peaks, which limits the number of fitting parameters. The black line shows the fit to the experimental data.

It ought to be mentioned that we have also considered the possibility of (NH_3_)_*n*_BzH^−^ ion generation. There are several arguments against it. First, even if these ions could account for the major peak, the next neighbouring peak could not be fully accounted for by the isotope contribution. Second, the (NH_3_)_*n*_BzH^−^ ion position would coincide with the (NH_3_)_*n*_Bz^−^ isotope position and could not thus explain the width of the major peak, which is consistently larger than the peak width of the (NH_3_)_*n*_Bz^−^ for all *n*. Third, we are analyzing the spectrum at 1.3 eV electron energy. The DEA of NH_3_ yielding H^−^ (or NH_2_^−^) is not possible at this energy. Thus, the energy to break an N–H bond would have to be provided by the localization of the solvated electron on Bz^−^, which is not sufficient, as will be discussed below. On top of all these arguments, we also provide an experimental proof for the metastable evaporation of NH_3_ molecule(s) from the cluster. Changing the middle grid potential of our reflectron TOF spectrometer, we could demonstrate that the position of the metastable peak shifts with respect to the peak displaced by Δ*m*/*z* = +1 from the (NH_3_)_*n*_Bz^−^ peak (see Fig. S2 in ESI†).

The right panel, Fig. 7(b), shows an analysis of the neighboring mass peak (NH_3_)_*n*_^−^ ions *n* = 47 without benzene. Here, the contribution of the metastable decay of the larger *n* = 48 cluster is small, probably within our signal-to-noise limit. Thus, the majority (NH_3_)_*n*_^−^ ions are stable during their flight time in our TOF. Only a small fraction of the order of a few percent of the (NH_3_)_*n*_^−^ ions could evaporate an NH_3_ molecule. On the other hand, the doped (NH_3_)_*n*_Bz^−^ ions are definitely metastable and evaporate ammonia monomers. This is further discussed below.

### 3.3 Metastable decay of doped clusters

**Fig. 8:**
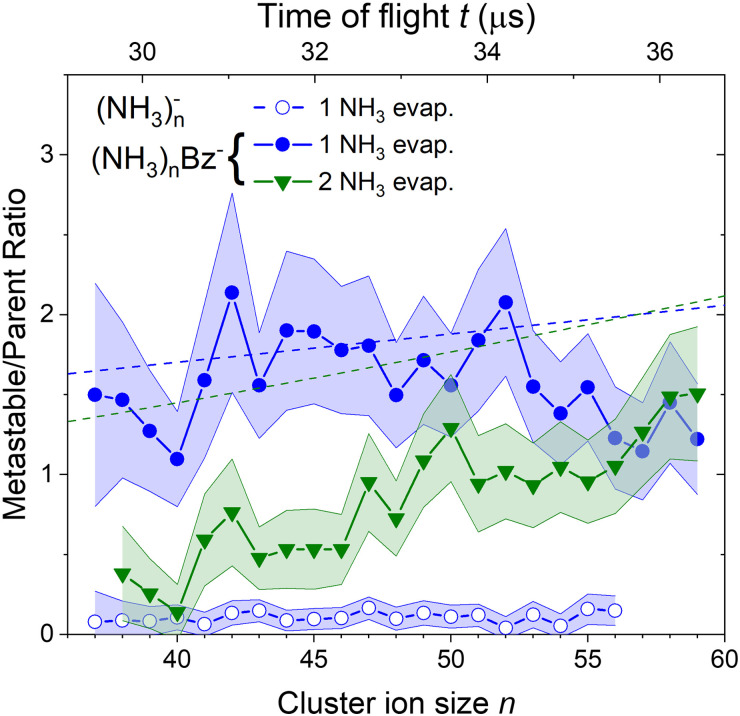
The metastable ratios (daughter/parent ion intensity ratio) evaluated from the mass spectrum in Fig. 6(b). Open symbols show the ratio for evaporation of one NH_3_ molecule from (NH_3_)_*n*_^−^ cluster ions. Closed symbols show the ratio for evaporation of 1 (blue) and 2 (green) NH_3_ molecules from the doped (NH_3_)_*n*_Bz^−^ clusters. Dashed lines show the metastable fragment ratios calculated assuming first order kinetics for the decay with a time constant of 18 μs (see the text for details).

To quantify the metastable decay of the (NH_3_)_*n*_Bz^−^ ions by NH_3_ evaporation, we evaluate the ratio of the daughter fragment peaks to their parent ions that remained stable. The evaluation essentially follows the procedure outlined above for the (NH_3_)_*n*_Bz^−^, *n* = 42, example in Fig. 7. However, due to the spectrum complexity and the number of free parameters, the analysis is simplified avoiding extensive spectra fitting. We integrate the mass peaks by simply summing the experimental intensities in the given peak area rather than fitting them with Gaussian. The isotope contributions are calculated based on the natural isotope ratios and subtracted/added from/to the corresponding integrals. The intensities of the two metastable peaks neighboring to a (NH_3_)_*n*_Bz^−^ peak are thus evaluated, and their ratios to the integrated intensities of the corresponding (NH_3_)_*m*_Bz^−^ peak with *m* = *n* + 1 and *m* = *n* + 2 are calculated. These ratios, shown in Fig. 8, correspond to the metastable fractions of (NH_3_)_*m*_Bz^−^ ions decaying by the evaporation of 1 (blue) and 2 (green) NH_3_ molecules. The analysis has been done for all the mass peaks with a sufficient signal-to-noise ratio in the spectrum in Fig. 6(b). Since the mass peaks are not fitted, their overlap is neglected in this evaluation, which can increase the error bars at large masses above *m*/*z* 1000 with an increasing width of the mass peaks and their overlap. Therefore, the larger masses have not been included, although the corresponding peaks appear in the mass spectrum.

The same procedure applied to the (NH_3_)_*n*_^−^ peaks without the adsorbed Bz molecules delivers no significant metastable fraction as is obvious from the detailed analysis of the *n* = 47 peak in Fig. 7(b). Nevertheless, we analyze the (NH_3_)_*n*_^−^ ion peaks in the spectrum shown in Fig. 6(b) by the same procedure and plot the metastable ratio obtained for evaporation of one NH_3_ molecule from (NH_3_)_*n*_^−^ cluster ions in Fig. 8 for comparison (open symbols).

A similar analysis for the spectrum at 6.0 eV is not possible, since there are too many overlapping contributions in this spectrum: There are contributions of both (NH_3_)_*n*−1_NH_2_Bz^−^ and (NH_3_)_*n*_Bz^−^ ions and their isotopes and all these ions contribute also to the metastable fraction. Thus, the analysis would be impossible without arbitrarily fixing some of the free parameters.

Before discussing the metastable fraction trends in Fig. 8, we consider the time, which the clusters spend in the first field-free region of the TOF. It depends on their size and for the (NH_3_)_*n*_Bz^−^ clusters *n* = 36 and 60 it corresponds to *t* ≈ 29.1 and 36.8 μs, respectively. Thus, the cluster ion size can be converted to the time window for NH_3_ molecule(s) evaporation, which is shown by the top axis in Fig. 8.

One might tend to interpret the structure on the metastable fraction dependency in Fig. 8 as corresponding to some lower/higher stability of the parent ions. Evaporation is a successive process: 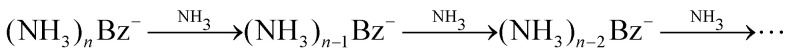. Thus, the local extremes for the evaporation of a single NH_3_ monomer should be reflected by similar extremes for the evaporation of 2 NH_3_ just shifted by Δ*n* = −1, which is not the case. Thus, the extremes in the metastable fraction rather correspond to fluctuations within error bars of our experiment and evaluation procedures.

**Fig. 9:**
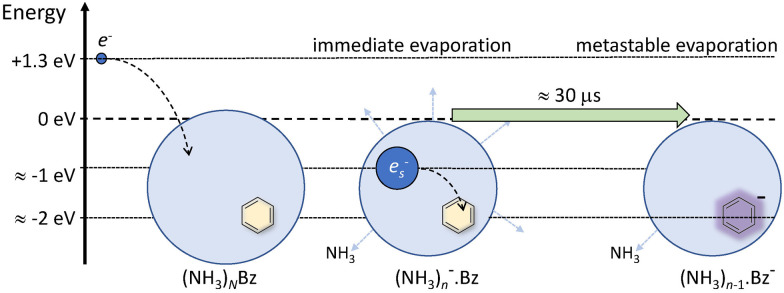
A schematic picture of electron attachment to (NH_3_)_*N*_·Bz clusters. A slow electron with a kinetic energy of about 1.3 eV generates a solvated electron in the cluster, which leads to immediate evaporation of NH_3_ molecules releasing the excess energy and stabilizing the cluster ion. Subsequently, the ion travels about 30 μs through the TOF field free region, and the electron can localize on benzene leading to NH_3_ molecule(s) evaporation due to the released Bz^−^ stabilization energy.

Nevertheless, the general trends in the dependency are unambiguous. The total metastable fraction ranging between 1 and 3 suggests that more than half of the benzene containing ions (NH_3_)_*n*_Bz^−^ in the size region *n* = 37–59 is metastable on the timescale of about 33 μs and decays by evaporating NH_3_ molecules. This is in strong contrast with the pure (NH_3_)_*n*_^−^ ions which are essentially stable in this time window. It ought to be stressed that we are discussing the cluster ions generated by 1.3 eV electron attachment, *i.e.*, presumably a solvated electron is initially formed in the cluster. In the case of the (NH_3_)_*n*_^−^ cluster ions, the solvated electron is formed in the cluster and eventually stabilized by immediate evaporation of NH_3_ molecules, and the stable (NH_3_)_*n*_^−^ ion is accelerated into the TOF. In Bz containing clusters, the solvated electron is initially formed as in the pure ammonia clusters, since the clusters are mainly composed of ammonia with only one benzene molecule. However, the solvated electron can migrate and be localized on benzene generating a Bz^−^ radical anion with a certain time delay. This process releases energy into the cluster, which leads to the metastable evaporation of NH_3_ molecules. The vertical binding energy (VBE) of the benzene radical anion in ammonia has been calculated as VBE = −2.3 eV.^18^ The VBE of a solvated electron in bulk ammonia corresponds to −1.25 eV.^4^ These values are different in smaller clusters (*e.g.*, VBE of an electron in (NH_3_)_*n*_^−^, *n* = 41, is 0.55 eV), however, their cluster size dependence is similar and thus their difference of 1.05 eV is roughly constant. This energy is released upon localization of the solvated electron on Bz^−^. It can be compared to the binding energy of an NH_3_ molecule in clusters. For neutral ammonia dimer it is about 0.14 eV,^32,33^ and it increases to 0.33 eV in the bulk.^32^ The binding energies for our finite size negatively charged clusters are probably in the same energy region. Thus, the released energy is sufficient to evaporate several NH_3_ molecule. The processes involved in the electron attachment to (NH_3_)_*N*_·Bz cluster are depicted schematically in Fig. 9.

We can compare the general trends in the metastable fraction for the evaporation of one and two NH_3_ molecules. The latter unambiguously increases as a function of the cluster size *n*, while the dependence of the metastable fraction for the evaporation of one NH_3_ molecule seems to decrease, although the decrease is within the error bars. Such behavior, could be expected from an analogy with the successive first order kinectics. An NH_3_ molecule evaporation can be assumed as an exponential process e^−*t*/*τ*^ with a certain lifetime *τ*. The fact that the two dependencies in Fig. 8 tend to cross suggests that the lifetime is comparable to about half of the mean time spent by the clusters in the TOF region, *i.e.*, *τ* ≈ 18 μs.

A more quantitative analysis yields the same evaporation time. It follows from a simple kinetic model that the evaporation of *k* molecules follows Poisson statistics 
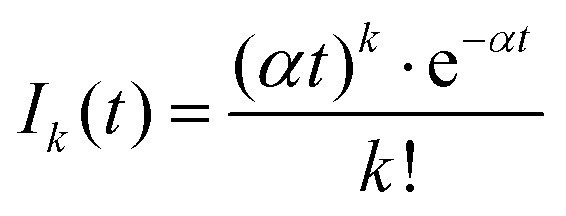
, where *α* = 1/*τ*. Thus we get for the parent (NH_3_)_*n*_Bz^−^ ion the exponential decay *P*^*n*^(*t*) = *P*^*n*^_0_e^−*αt*^, and for the daughter fragments after evaporation of one and two NH_3_ units *D*^*n*^_1_(*t*) = *P*^*n*^_0_*αt*e^−*αt*^ and 
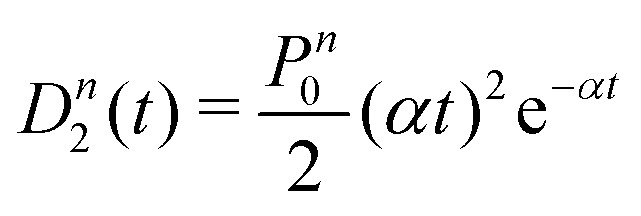
, respectively. The ratios of the metastable clusters, from which one and two molecules evaporated *D*^*n*^_1_/*P*^*n*^ and *D*^*n*^_2_/*P*^*n*^ are shown by the dashed lines in Fig. 8. The above mentioned conversion of the cluster size *n* to the time *t* spent in the first field-free region of the TOF allows us to compare the metastable ratios obtained for different cluster sizes directly with the calculated ones. In the calculation, we have varied the evaporation time *τ* as a free parameter in order to obtain the intercept between the theoretical dependence for the evaporation of 1 and 2 molecules at approximately 35.5 μs, where the intercept of the experimentally determined ratios appears, *i.e.*, corresponding to Bz·(NH_3_)_*n*_^−^, *n* ≈ 57. The agreement of the intercepts has been obtained for *τ* ≈ 18 μs as estimated qualitatively above.

Although the agreement between the experimentally measured metastable fractions and the kinetic model calculation is not quantitative, the trends agree and the obtained mean evaporation time is reasonable. Thus, we can claim that kinetic information about the cluster evaporation could be obtained from the cluster mass spectrum. Indeed, the observation of cluster metastable evaporation in the mass spectra was analyzed already in the early works by Castlemans group.^30^ However, a new dimension has been added here by the conversion of the cluster size into the time axis allowing a direct comparison with the decay curves (in the time domain) for evaporation of one and two molecules based on a simple first-order kinetic model. The new method is limited by the signal to noise ratio and overlap of the metastable mass peaks and also by their complex analysis. The most limiting factor represents the very short time window given by the range of cluster sizes, for which the analysis was possible.

## 4 Conclusions

We investigate electron attachment to large ammonia (NH_3_)_*N*_, *N̄* ≈ 320, clusters. The following conclusions can be drawn from our TOF mass spectrometric experiment:

• The negative ion spectra exhibit (NH_3_)_*n*−1_NH_2_^−^ and (NH_3_)_*n*_^−^ cluster ion fragments.

• Two peaks are observed in the negative ion yield: one at the electron energy of 6.0 eV and one at low energies, possibly at 1.3 eV.

• At 6 eV electron energy the (NH_3_)_*n*−1*n*−1_NH_2_^−^ ions are produced in agreement with the DEA of an isolated NH_3_ molecule. The (NH_3_)_*n*_^−^ ions are formed at this energy as well by fragment caging after the DEA.

• At 1.3 eV only the (NH_3_)_*n*_^−^ ions are populated. The observed threshold size *n* = 35 for the appearance of these ions in the mass spectrum measured at 1.3 eV roughly agrees with the threshold size *n* = 37, at which the low energy peak at 1.3 eV starts appearing in the ion yield measurements. The (NH_3_)_*n*_^−^ ions observed at 1.3 eV are interpreted being due to the solvated (ammoniated) electrons.

However, the major focus of the present investigation is on the ammonia clusters doped with benzene molecules (NH_3_)_*N*_Bz. Here, we observe the following:

• The negative ion spectra exhibit (NH_3_)_*n*−1_NH_2_Bz^−^ and (NH_3_)_*n*_Bz^−^ cluster ion fragments in analogy to the pure ammonia clusters.

• Also the energy dependent negative ion yield corresponds to pure ammonia clusters with the two peaks at 6.0 eV and 1.3 eV.

• The (NH_3_)_*n*_Bz^−^ (and possibly also (NH_3_)_*n*−1_NH_2_Bz^−^) cluster ions exhibit a strong metastable decay in the first field free region of our TOF. A detailed analysis of the (NH_3_)_*n*_Bz^−^ ions between *n* = 37 and 59 revealed that more than half of them evaporate one or two NH_3_ molecules in the time window ≈30–36 μs.

• The lifetime for NH_3_ evaporation from these clusters was evaluated as *τ* ≈ 18 μs.

• The instability of the doped (NH_3_)_*n*_Bz^−^ cluster ions is in strong contrast to the stable (NH_3_)_*n*_^−^ ions. We suggest that it is caused by the attachment of a solvated electron on benzene generating solvated benzene radical anion Bz^−^. This localization and stabilization leads to an energy release leading to the NH_3_ molecule(s) evaporation. It validates the recent theoretical calculations.^18^

There are two outcomes worth to stress: First, in terms of a practical importance, the behavior of a solvated electron in benzene doped ammonia clusters is relevant to the Birch reduction process, and we provide a molecular-level insight into the dynamics of the transition of the solvated electron to the solvated Bz^−^ radical anion. Second, the determination of the evaporation time from our mass spectrometric experiment is enabled by the measurement of metastable cluster fraction for different cluster sizes, which can be converted to different time windows for the evaporation. This actually represents a new method, although quite limited in the present case by the very short time window given by the range of cluster sizes, for which the analysis is possible. Nevertheless, it could be exploited for suitable systems in future studies.

## Author contributions

M. F.: conceptualization, methodology, writing – original draft, review and editing; A. P.: investigation, formal analysis, visualization; S. B.: investigation, formal analysis, visualization; P. S.: writing – review and editing.

## Conflicts of interest

There are no conflicts to declare.

## Supplementary Material

CP-024-D2CP02979K-s001
