# Supplementary material for: Stabilization of benzene radical anion in ammonia clusters
Source: Phys Chem Chem Phys. 2022 Nov 2;24(44):27128–35. doi: 10.1039/d2cp02979k (PMC9673686; doi:10.1039/d2cp02979k)
Supplement: CP-024-D2CP02979K-s001 [file CP-024-D2CP02979K-s001.pdf]

Electronic Supplementary Material (ESI) for Physical Chemistry Chemical Physics.
This journal is © the Owner Societies 2022

ELECTRONIC SUPPLEMENTARY INFORMATION

Stabilization of benzene radical anion in ammonia clusters

Andriy Pysanenko,[a] Stefan Bergmeister,[b] Paul Scheier,[b] and Michal Fárník*[a]

[a] *J. Heyrovský Institute of Physical Chemistry, v.v.i., The Czech Academy of Sciences, Dolejškova 2155/3, 182 23 Prague, Czech Republic. Fax: +420 2 6605 3910; Tel: +420 2 6605 3206; E-mail: michal.farnik@jh-inst.cas.cz*

[b] *Institut für Ionenphysik und Angewandte Physik, Universität Innsbruck, Technikerstr. 25, A-6020 Innsbruck, Austria.*

Mass Spectra

Mass spectra of positively and negatively charged pure ammonia clusters of the mean size $\bar{N} \approx 320$ corresponding to **Fig. 1** in the main article are shown in more details in **Fig. S01**. The assignment of the major ion peaks is indicated. The experimental conditions and further details are outlined in the main article.

Fig. S02 shows a detail section of the mass spectra of negatively charged ammonia clusters doped with benzene molecules, which are shown in the main article in **Fig. 6**. The $(NH_3)_n^-$ series is labeled in the top spectrum measured at the electron energy of 6.0 eV and the same series is obvious in the bottom spectrum. The benzene doped $Bz\cdot(NH_3)_n^-$ cluster ions are labeled in the bottom spectrum (similar series is indeed observed in the top spectrum), however, the doped clusters are dominated by metastable decay, as explained in the main article.

Fig. S03 shows the positive ion mass spectra of ammonia $(NH_3)_N$ clusters doped with benzene (Bz) molecules at different pickup pressures. The spectrum illustrates that under our current experimental conditions outlined in the main article corresponding to the top spectrum at the pickup pressure of 2.5×10^{-5} mbar, the doped clusters contain prevailingly only a single NH_3 dopant molecule. The abundances of $Bz_2(NH_3)_nH^+$ clusters containing two Bz units are negligible compared to the ones with a single unit $Bz_2(NH_3)_nH^+$. Only at 4-times higher pickup pressure of 10×10^{-5} mbar the $Bz_2(NH_3)_nH^+$ series becomes clearly observable (bottom spectrum). It is worth noting that in the spectrum of negatively charged clusters $(NH_3)_nBz^-$, the cluster ions with two Bz units could not be identified.

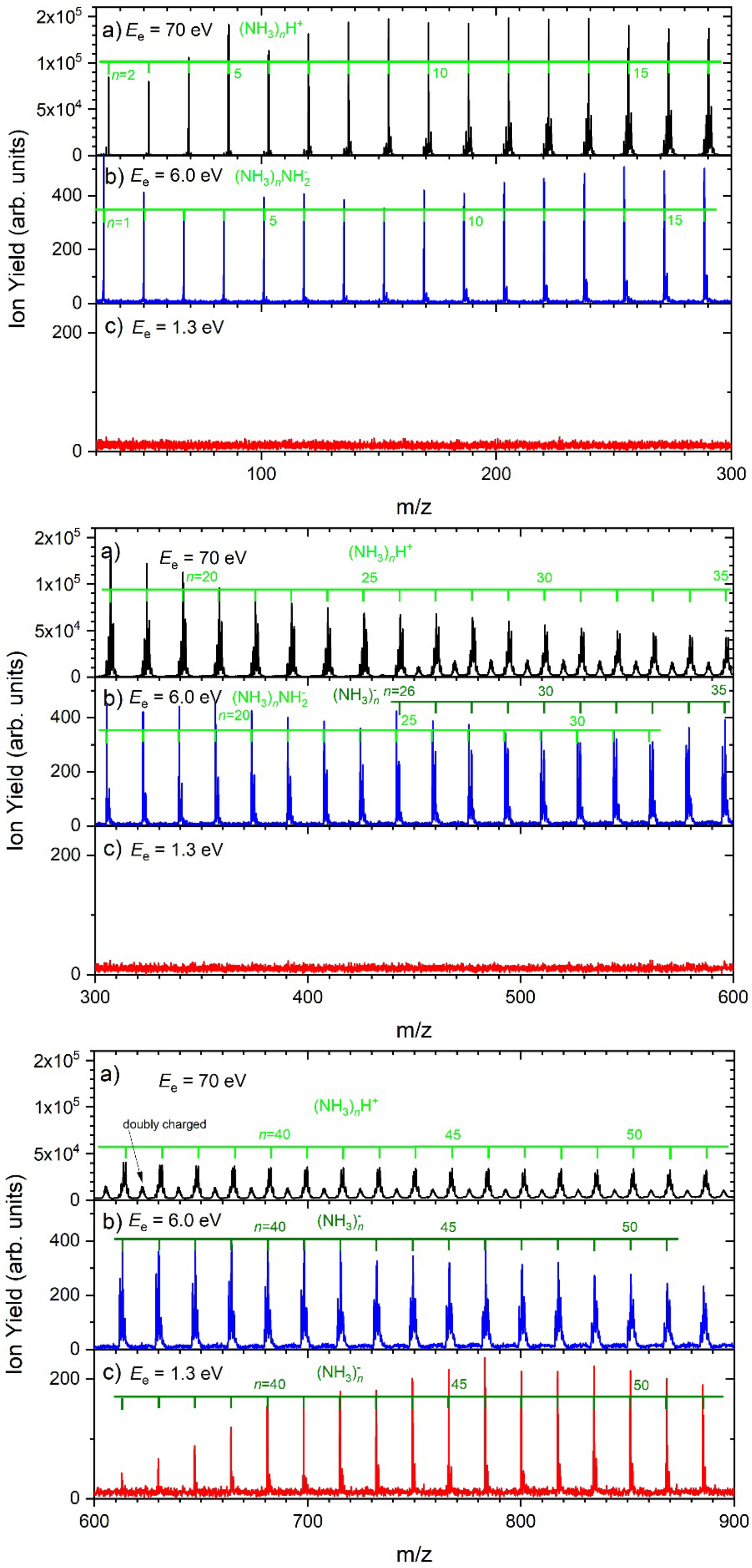

Fig. S01: *Mass spectra of positively and negatively charged pure ammonia clusters.*

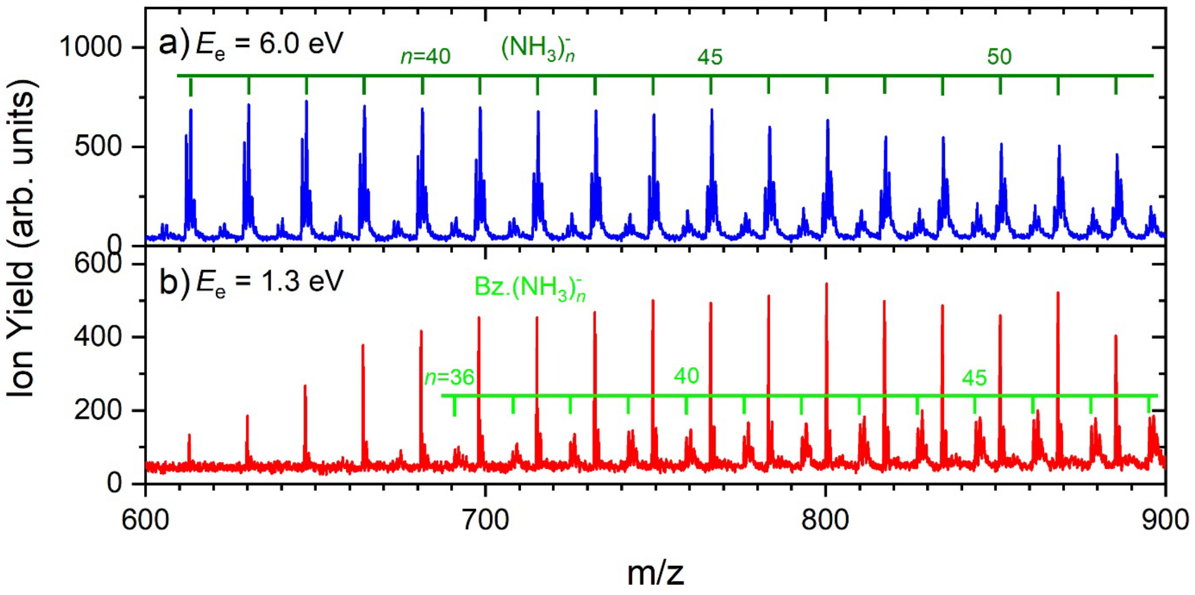

Fig. S02: *Mass spectra of negatively charged ammonia clusters doped with benzene molecules.*

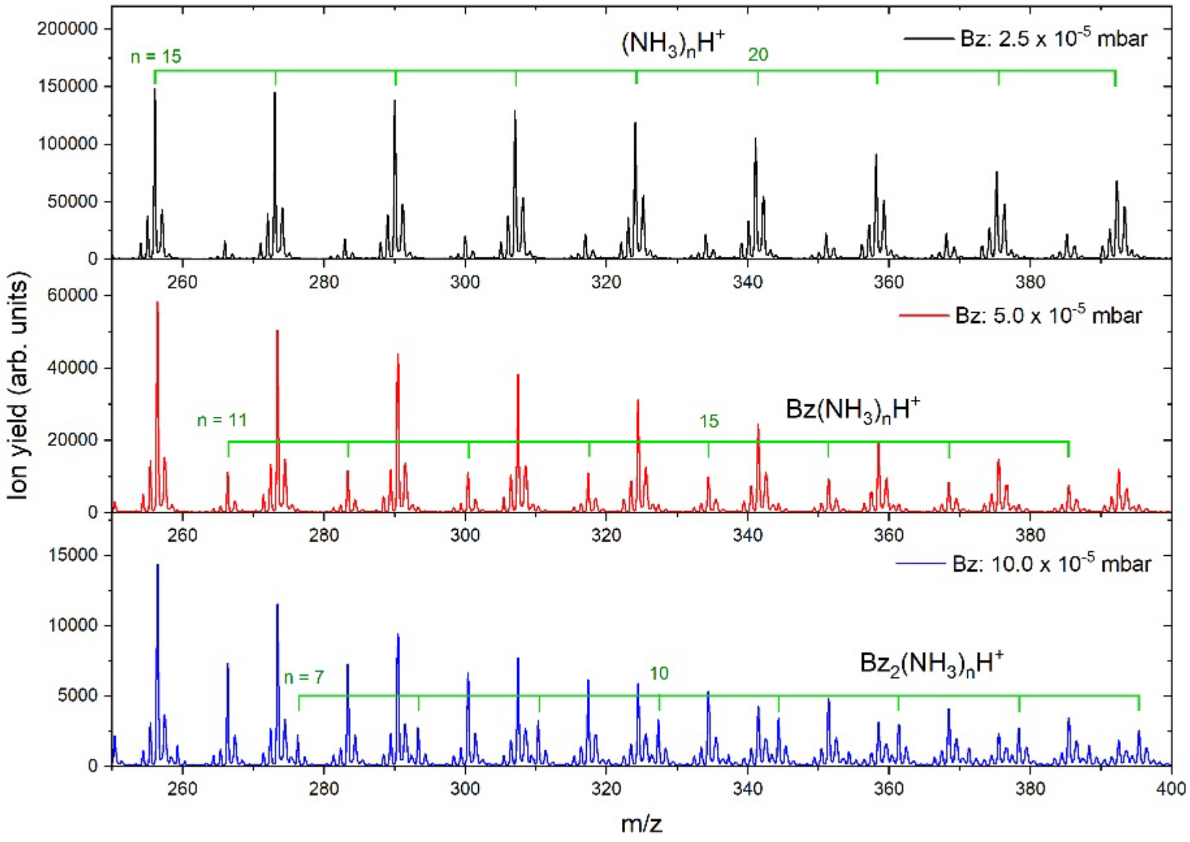

Fig. S03: *Evidence for singly doped ammonia clusters in the positive ion spectrum.*

Isotope Contribution

In large clusters, isotope contributions can play an important role for mass peak assignments. In our present case the ^{15}N isotope (apart from the most abundant ^{14}N isotope) has to be considered with a natural abundance corresponding to $a \approx 0.004$. The probability $P_k{}^n$ to find k isotopes ^{15}N in a cluster ion containing n ammonia molecules follows binomial distribution:

$$P_k^n = \binom{n}{k}(a)^k(1-a)^{n-k} = \frac{n!}{k!\,(n-k)!}(a)^k(1-a)^{n-k}$$

For example, in $(NH_3)_nH^+$ cluster ion containing n = 40 ammonia molecules the probability of finding ^{15}N isotope corresponds to 14%. Therefore, next to the mass peak of this ion at m/z = 682 there is the isotope peak at 683 with abundance of about 14% of the main peak. A useful web-tool can be implemented to calculate isotope contributions.[1]

Metastable Decay

A reflectron TOF mass spectrometer can be used to determine a metastable decay of clusters. The methodology can be found, e.g., in earlier studies of metastable decay of ammonia and water clusters and references cited therein,[2,3] and also in basic literature about TOF mass spectrometers, e.g. Ref. 4. A brief explanation is given in the main article on page 4, where we argue that a mass peak assigned to $(NH_3)_n^-$ clusters has actually three contributions: the stable $(NH_3)_n^-$ ions, the fragments from $(NH_3)_{n-1}NH_2^- \leftarrow (NH_3)_nNH_2^-$ metastable decay, and the isotope contribution of the stable $(NH_3)_{n-1}NH_2^-$ ions. Similarly, the next smaller peak following the $(NH_3)_n^-$ ion contains the isotope contribution and also a contribution from the metastable decay of $(NH_3)_{n+1}NH_2^-$ ion where two NH_3 molecules are successively evaporated. This is indicated for a particular example of n = 28 in **Fig. S04** by the black dashed arrows.

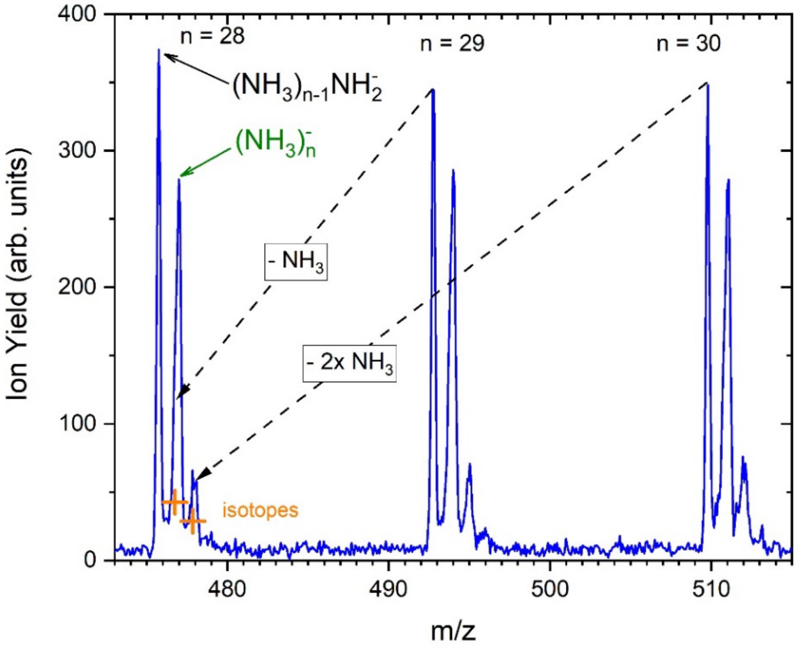

Fig. S04: *Illustration of the contribution from metastable decays of $(NH_3)_nNH_2^-$ ions in the mass spectra*

Fig. S05 illustrates the geometrical arrangement and voltages in our reflectron TOF mass spectrometer (note that the voltages are negative as used for positive ion detection; for negative ions opposite polarities are used). Using these voltages and geometry, time of flight for different stable and metastable ions can be calculated. We have recently performed comprehensive experiments with positively and negatively charged water clusters, where their metastable behavior in our TOF was carefully analyzed and compared to previous works on water clusters.[2] This allows us to calibrate and confirm our calculations of flight times of different stable and metastable ions in our TOF, and compare them with our present TOF spectra for ammonia clusters.

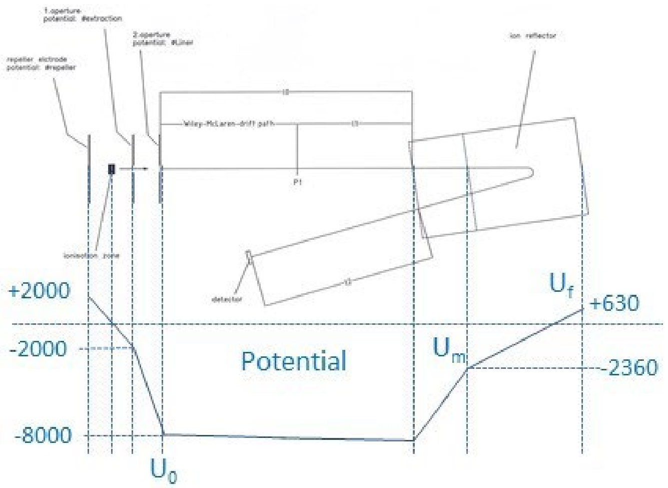

Fig. S05: *Illustration of the geometrical arrangement and voltages in our reflectron TOF*

Table 1: Calculated flight times for selected ions in our reflectron TOF mass spectrometer

Ion	*m/z*	Time of flight τ (μs)
$(NH_3)_{27}NH_2^-$	475.736	24.2213
$(NH_3)_{28}^-$	476.743	24.2467
Metastable NH_3 evaporation $(NH_3)_{27}NH_2^- \leftarrow (NH_3)_{28}NH_2^-$	475.736	24.2593
Metastable $2{\times}NH_3$ evaporation $(NH_3)_{27}NH_2^- \leftarrow (NH_3)_{29}NH_2^-$	475.736	24.2732
Hypothetical: $(NH_3)_{28}H^-$	477.751	24.2721
$(NH_3)_{28}NH_2^-$	492.762	24.6500
$(NH_3)_{29}NH_2^-$	509.789	25.0714

For the particular example of the mass peaks around the ion $(NH_3)_n^-$, $n = 28$, illustrated above we provide the calculated flight times in **Tab. 1**. From these flight times, it is obvious that the $(NH_3)_{27}NH_2^-$ cluster ions after the metastable evaporation of one NH_3 molecule arrive at the detector with a delay $\Delta\tau$ less than 13 ns after the stable $(NH_3)_{28}^-$ ion; this corresponds to less than 1 mass unit in the mass scale. Thus, the stable $(NH_3)_{28}^-$ ion and the metastable $(NH_3)_{27}NH_2^-$ ion

after the evaporation of NH_3 from $(NH_3)_{28}NH_2^-$ overlap in the mass spectrum as indicated in **Fig. S04**. Similarly, the arrival time of the metastable ion after evaporation of two NH_3 molecules $(NH_3)_{27}NH_2^- \leftarrow (NH_3)_{29}NH_2^-$ arrives close to hypothetical $(NH_3)_{28}H^-$ ion.

Indeed, due to stray fields and inexact geometry, the calculated flight times are not exact and needed to be calibrated. However, the metastable ions can be proved by changing voltages in the reflectron on our TOF mass spectrometer. The method has been again described in details in the above mentioned publications.[2,3] Essentially, by changing the voltages, the metastable peaks shift in the mass scale, while the stable ions remain at their corresponding masses. This is illustrated below for an example of a doped $(NH_3)_{n+1}Bz^-$ cluster ion.

The $(NH_3)_nBz^-$ cluster ion that originates from $(NH_3)_{n+1}Bz^-$, from which a monomer NH_3 unit evaporated in the first field free region, arrives at the detector at a time close to a stable $(NH_3)_nBz^-$ cluster ion. Under the focusing condition of our TOF, the displacement of this $(NH_3)_nBz^-$ fragment ion almost exactly corresponds to $\Delta m/z$ = +1 from the stable $(NH_3)_nBz^-$ ion as illustrated above for the undoped clusters. Thus the $(NH_3)_nBz^-$ fragment from the metastable $(NH_3)_{n+1}Bz^-$ decay overlaps with the isotope contribution of the stable $(NH_3)_nBz^-$ ions. However, the metastable peak can be distinguished from the isotope by changing the middle grid potential U_g of our reflectron. In the mass calibrated scale, the metastable fragment peak position changes as a function of U_g, with respect to the stable $(NH_3)_nBz^-$ ion while the stable $(NH_3)_nBz^-$ ion position remains fixed.

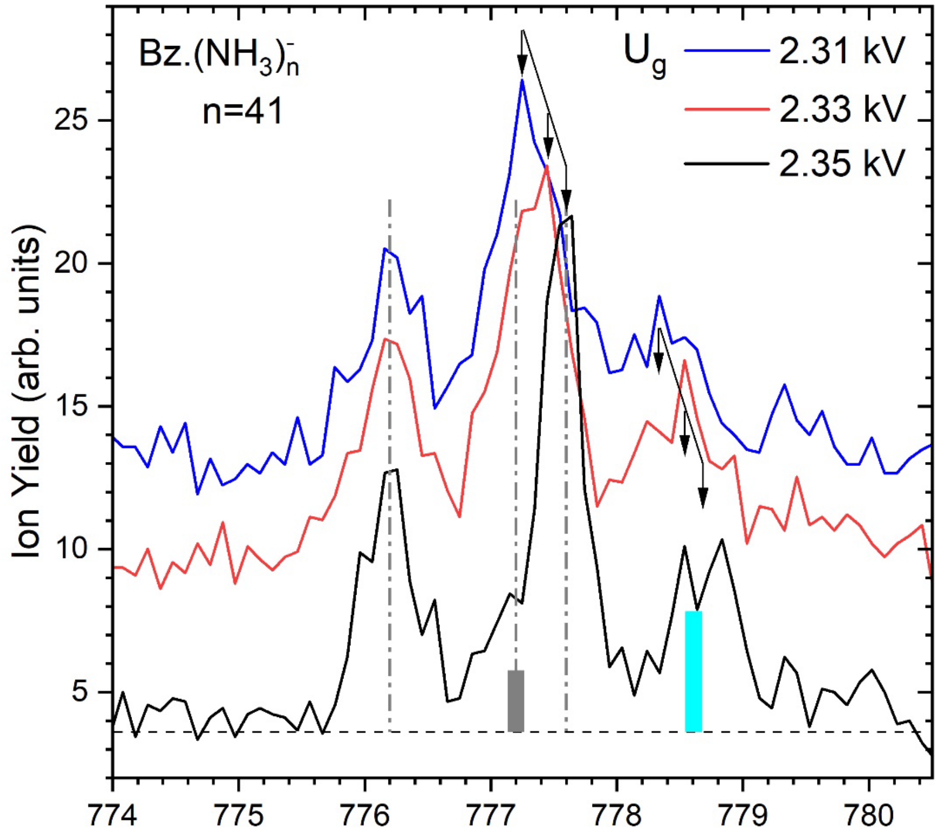

Fig. S06: *Experimental evidence for the metastable evaporation of NH_3 molecule(s) from the clusters.*

This is illustrated in **Fig. S06** for the example of n = 41 cluster. The first smaller peak at *m/z* = 776.2 corresponds to the $(NH_3)_nBz^-$, n = 41, cluster ion that did not evaporate any NH_3 units in the TOF. The corresponding isotope contribution at *m/z* = 777.2 is indicated by a grey bar. The cyan bar corresponds to the isotope contribution of the metastable peak. Clearly, the actual mass peak is significantly higher and shifted in its position to a higher *m/z* = 777.6. More importantly, its position changes with U_g as indicated by small arrows at the top. Therefore, this peak cannot be assigned to the isotope contribution nor to a hypothetical $Bz(NH_3)_nH^-$ ion. It corresponds to the fragment ion of $(NH_3)_nBz^-$, n = 42, from which one NH_3 unit evaporated during its flight in the first field free region. This assignment is backed by the next smaller peak at *m/z* = 778.6. Its abundance roughly corresponds to the isotope of the major peak, i.e., the metastable fragment $(NH_3)_nBz^-$, $n = 41 \leftarrow 42$, as indicated by the higher gray bar, and its position follows the main peak with changing U_g. Thus this peak mainly corresponds to the isotope of the metastable $(NH_3)_nBz^-$, $n = 41 \leftarrow 42$ fragment ion.

It ought to be mentioned that we have recently done a very extensive investigation of metastable decay of positively and negatively charged large water clusters,[5] which enabled us to calibrate theoretically calculated flight times of cluster ions in our TOF mass spectrometer for various scenarios of metastable evaporation of monomer units. These calculations agree reasonably well with the metastable peak positions in the present TOF spectra (in the time domain) as already outlined above. More importantly the relative shift with U_g in the metastable peak position in time is well reproduced by the calculations. This further confirms our assignment of the metastable fragment ions.

References

[1] https://www.sisweb.com/mstools/isotope.htm

[2] S. Q. Wei and A. W. Castleman, Using reflectron tofms techniques to investigate cluster dynamics and bonding, Int. J. Mass Spectrom. Ion Processes 131, 233 (1994).

[3] L. Belau, K. R. Wilson, S. R. Leone, and M. Ahmed, Vacuum ultraviolet (VUV) photoionization of small water clusters, J. Phys. Chem. A 111, 10075 (2007).

[4] U. Boesl: Time-of-flight mass spectrometry: introduction to the basics, Mass Spectrometry Reviews, 36, 86 (2017)

[5] *work in progress*.
